# Supplementary material for: Qualitative Focus Groups with Professionals of Special Education and Parents of Young Females with Intellectual Disability Exploring Experiences with Menstrual Hygiene Management and the Trigger for the Non-Therapeutic Hysterectomy in Mexico
Source: Healthcare (Basel). 2022 Sep 4;10(9):1690. doi: 10.3390/healthcare10091690 (PMC9498658; doi:10.3390/healthcare10091690)
Supplement: Supplementary file 1 [file healthcare-10-01690-s001.zip › healthcare-1849305-supplementary.pdf]

S1 Appendix. Outline of the topic guide

The following is a topic guide used to prompt participants to openly convey their viewpoints regarding the menstrual hygiene management and hysterectomy. It was not intended that each of the following questions be asked word by word, but rather that they be used as a memory aid to ensure that all important areas would be covered.

Start interview for all participants

Introduction of the moderator, the study, and its purpose.

Estimated duration of the interview.

Consent and confidentiality: written consent, audio recording, how the data will be used.

Possibility to stop at any time, or decline to answer any questions without any consequences.

Background for all participants

Demographic data

Focus group discussions

The moderator often started the focus group by asking: can you tell me about menstruation?

Although participants individually answer the facilitator's questions, they were encouraged to talk and interact each other. When necessary, prompts were used to explore and clarify individual and share perspectives; and, also, to cover the following points:

- How did you (as a primary carer) learn about menstrual hygiene management?
- What do you (as a primary carer) do that your daughter carry out your menstrual hygiene management indications?
- How did your daughter learn about what is happening?
- Did you involve your daughter in the menstrual hygiene management?

- How much did all the received information in the school improve your understanding (as a primary carer) of the menstrual hygiene management and the health status of your daughter?
- How do you think about contraceptive methods to and pregnancies in females with ID?
- Did you involve your daughter in the decision-making process?
- What aspects of context, attitudes, and experience, facilitate or constrain the menstrual hygiene management?
- How do you see yourself in the near future?

The moderator closed the focus group by asking: is there a missing issue to talk about? and by reaching consensus on conclusions.
